# Biofilm-Producing Bacteria and Risk Factors (Gender and Duration of Catheterization) Characterized as Catheter-Associated Biofilm Formation

**DOI:** 10.1155/2021/8869275

**Published:** 2021-02-22

**Authors:** Wani Devita Gunardi, Anis Karuniawati, Rainy Umbas, Saptawati Bardosono, Aida Lydia, Amin Soebandrio, Dodi Safari

**Affiliations:** ^1^Department of Clinical Microbiology, Faculty of Medicine, Krida Wacana Christian University, Jakarta 1151, Indonesia; ^2^Department of Clinical Microbiology, Faculty of Medicine, Universitas Indonesia, Jakarta 10320, Indonesia; ^3^Department of Urology, FMUI-CMH, Jakarta 10430, Indonesia; ^4^Department of Nutrition, Faculty of Medicine, Universitas Indonesia, Jakarta 10430, Indonesia; ^5^Department of Internal Medicine, Faculty of Medicine, Universitas Indonesia, Jakarta 10430, Indonesia; ^6^Eijkman Institute for Molecular Biology, Jakarta 10430, Indonesia

## Abstract

**Background:**

A catheter-associated urinary tract infection (CA-UTI) is preceded by biofilm formation, which is related to several risk factors such as gender, age, diabetic status, duration of catheterization, bacteriuria before catheterization, virulence gene factor, and antibiotic usage.

**Aims:**

This study aims to identify the microbial composition of catheter samples, including its corresponding comparison with urine samples, to determine the most important risk factors of biofilm formation and characterize the virulence gene factors that correlate with biofilm formation.

**Methods:**

A longitudinal cross-sectional study was conducted on 109 catheterized patients from September 2017 to January 2018. The risk factors were obtained from the patients' medical records. All catheter and urine samples were cultured after removal, followed by biomass quantification. Isolate identification and antimicrobial susceptibility testing were performed using the *Vitex2* system. Biofilm-producing bacteria were identified by the *Congo Red Agar* (CRA) method. A PCR test characterized the virulence genes of dominant bacteria (*E. coli*). All data were collected and processed for statistical analysis.

**Results:**

Out of 109 catheterized patients, 78% of the catheters were culture positive, which was higher than those of the urine samples (37.62%). The most common species isolated from the catheter cultures were *Escherichia coli* (28.1%), *Candida* sp. (17.8%), *Klebsiella pneumoniae* (15.9%), and *Enterococcus faecalis* (13.1%). *E. coli* (83.3%) and *E. faecalis* (78.6%) were the main isolates with a positive CRA. A statistical analysis showed that gender and duration prior to catheterization were associated with an increased risk of biofilm formation (*p* < 0.05).

**Conclusion:**

*E. coli* and *E. faecalis* were the most common biofilm-producing bacteria isolated from the urinary catheter. Gender and duration are two risk factors associated with biofilm formation, therefore determining the risk of CAUTI. The presence of PapC as a virulence gene encoding pili correlates with the biofilm formation. Biofilm-producing bacteria, female gender, duration of catheterization (more than five days), and PapC gene presence have strong correlation with the biofilm formation. To prevent CAUTI, patients with risk factors should be monitored by urinalysis tests to detect earlier the risk of biofilm formation.

## 1. Introduction

Urethral catheterization is a common procedure among hospitalized patients who must be bedridden for a period because of a severe illness, paralytic syndrome, or major surgery [1, 2]. One major problem which commonly causes patients to deteriorate from their current state is a catheter-associated urinary tract infection (CAUTI) [1, 3]. This condition could prolong the length of stay in the hospital, increase the morbidity and mortality rate, and cause a significant financial burden for the patients, their families, and the healthcare system [3–5].

CAUTI is a nosocomial infection, with the most common etiologies being Enterobacteriaceae such as *Escherichia coli* and *Klebsiella* sp. However, in a healthcare setting, *Pseudomonas aeruginosa* and yeast have a higher prevalence [1–5]. Those bacteria can form a biofilm. Biofilm formation is the first step to CAUTI pathogenesis [1, 2]. Biofilm bacteria have different behaviors compared to their planktonic state, which increases their virulence and resistance to antibiotics [1–4]. The biofilm can be monomicrobial or polymicrobial. Bacteria in biofilm ascend through the catheter into the bladder within 1 to 3 days and cause an infection [4].

Some bacterial virulence factors play an important role in the pathogenesis of biofilm formation. This virulence factor, such as the adhesin factor named Fimbriae type 1 (FimA), which plays a role in inducing adhesion to host epithelial cells, is an important factor in the early stages of biofilm formation as well as PapC, which forms pili formation to attach to host cells or catheter materials [6]. Fimbriae S (SfaS) also can bind to the upper and lower urinary tract epithelium (kidneys and bladder), allowing colonization to occur [7].

Several studies have also reported that age, gender, comorbid diseases, and duration of catheterization are risk factors for CAUTI. Geriatric patients may have a higher prevalence of CAUTI due to a declining immune system [3, 4, 8]. Female patients are more susceptible to CAUTI [2, 9], and CAUTI is more prevalent in diabetic patients [5, 10]. The long duration of catheterization poses a higher risk of infection in patients. Almost 26% of patients who have indwelling catheters for 2 to 10 days develop bacteriuria, and virtually all patients catheterized for one month develop bacteriuria [5, 11]. The increased duration of catheterization is also a risk factor of biofilm formation in an indwelling catheter [12–14]. Therefore, this study aims to identify and characterize biofilm, determine the forming bacteria, and examine the correlation between patients' risk factors and the biofilm formation in a urethral catheter and characterize the virulence genes that are associated with biofilm formation.

## 2. Materials and Methods

### 2.1. Ethical Approval of the Study Protocols

Each study participant gave written informed consent under protocol 523/UN2.F1/ETIK/2017, as approved by the Ethics Committee of the Faculty of Medicine, University of Indonesia. All data were analyzed anonymously.

### 2.2. Patient and Sample Collection

The cross-sectional longitudinal study was conducted between September 2017 and January 2018 with an inpatient setting at a hospital in Tangerang, Banten, Indonesia. One hundred and nine patients were taken as the sample. The inclusion criteria were all adult patients (over 18 years old) receiving urethral catheterization for more than two days during hospitalization. The exclusion criteria were patients with underlying problems such as hydronephrosis and pyelonephritis, pregnancy, malignancy or immunocompromised diseases, and an allergic reaction to urethral catheter components. A sample was dropped out if the urinalysis and cultures were incomplete or if a patient refused to participate in this study. Data regarding the patients' age, gender, diabetic status, antibiotic usage, catheterization duration, and urinalysis results were obtained from medical records.

After the catheter was used and removed, urine was collected for a urinalysis to determine the presence of bacteriuria and be examined from the culture. After removal, the catheter's tip was aseptically cut for 5 cm in length and further cut into 1 cm five small pieces, which were then put in a sterile saline solution.

### 2.3. Catheter and Urine Preparation

The catheter was removed from the saline solution and rinsed using sterile aquades twice. Then, it was put inside a container with 5 ml of 10x phosphate-buffered saline (PBS) and went through sonification for 5 minutes at 25°C by sonicator (Bandelin® Sonorex Digitec) at 40 ± 5 kHz to get a bacterial suspension. Bacterial suspension from the catheter was used for the culture and biofilm test.

The urine from the catheter did not need special preparation for the culture. One hundred microliters (*μ*l) of urine was prepared for the culture to increase the ability to isolate bacteria from urine.

### 2.4. Catheter and Urine Cultures

The bacterial suspension isolated from 109 catheters and urine of catheterized patients were cultured using blood agar, chocolate agar, and MacConkey agar at 37°C for 24 hours. If there was bacterial growth in those mediums, the test continued with the identification of bacterial species.

### 2.5. Bacterial Identification

A single colony from the positive culture was picked up and inoculated into a Vitek 2 cartridge (Vitek 2 Compact, BioMerieux, France) according to the manufacturer's instructions to identify the bacterial species.

### 2.6. Biofilm-Producing Bacteria Identification

A Congo Red Agar (CRA) test was used to identify the biofilm-producing bacteria in the catheter sample. The CRA medium contained brain heart infusion broth (BHIB) (37 g/L), sucrose (50 g/L), agar-based No. 1 (10 g/L), and Congo Red (8 g/L). The CRA medium's preparation was done by adding HIB, sucrose, and agar in 800 mL of aquades in one place, and Congo Red in 200 mL of aquades in another place. Sterilization was done at 12°C for 15 seconds for each component. After the sterilization and the medium's temperature reached 55°C, Congo Red was added to the medium. Then, the medium was separated into several plates and left to cool until it became solid.

The CRA test was conducted by inoculating bacterial isolates in a CRA medium and incubating it at 35–37°C for 24 hours in an aerobic condition. The positive result showed a black colony with mucoid, a rough and crystalized consistency. *Escherichia coli* (ATCC 35218) was used as a positive control, while *Staphylococcus epidermidis* (ATCC 12228) and sterile CRA were used as a negative control in this study.

### 2.7. Biofilm Quantification

Biomass quantification was performed utilizing the collected samples obtained from catheters and using the method described by Balasubramanian et al. [15] with some modifications. The suspension which resulted from sonification was then filtered using a preweighed filter paper (initial weight) and 0.22 *μ*m pore size. After the filtration was completed, the filter paper was weighed to obtain the final weight. The whole weighing process was done using a moisture balance. The dry weight of the biomass was calculated using the following formulation:

(1) $$\begin{array}{c} \frac{\text{final weight}-\text{initial weight}}{\text{catheter surface area }\left({\text{cm}}^{2}\right)}. \end{array}$$

The catheter surface area was calculated using the following formulation:

(2) $$\begin{array}{c} \left(\text{catheter length}\times \text{outer diameter}\right)+\left(\text{catheter length}\times \text{inner diameter}\right)\text{ with the result in }{\text{cm}}^{2}. \end{array}$$

### 2.8. DNA Extraction

After incubation, 1 to 2 ml of medium with *E. coli* was centrifuged at 10,000 RPM for 1 minute. The supernatant was discarded. It was followed by adding the GA buffer and mixing it with Vortex. After that, 20 *μ*L of proteinase K was added and mixed with Vortex to become homogenous. Then, 220 *μ*L of GB buffer were added, mixed with Vortex, and incubated for 10 minutes at 70°C.

Absolute ethanol was then added and mixed to become homogenous. Then the mixture was centrifuged for 30 seconds at 12,000 RPM. The supernatant was then discarded, and 500 *μ*L of GD buffer was added and centrifuged for 30 seconds at 12,000 RPM. The supernatant was then discarded, and 700 *μ*L of PW buffer was added and centrifuged for 30 seconds at 12,000 RPM. The supernatant was then discarded, and 500 *μ*L of PW buffer was added and centrifuged for 30 seconds at 12,000 RPM. The supernatant was discarded, and the precipitate was then centrifuged for 2 minutes at 12,000 RPM to dry out the cell membrane. The precipitate was then placed in a new tube, and 50 *μ*L of TE buffer was added. The mixture was then incubated for 5 minutes at 37°C and then centrifuged for 2 minutes at 12,000 RPM. The DNA was then stored at −80°C before the next step.

### 2.9. Gene Identification

The identification of PapC, FimA, and SfaS genes used a polymerization chain reaction (PCR). In a tube, 10 *μ*L of master mix (quick tag TOYOBO) was combined with 8.2 *μ*L of nuclease-free water. Then, 1 *μ*L of DNA was added to the mixture. The mixture was then spun down before being added to the PCR equipment. The PCR program for all genes is listed in Table 1.

### 2.10. Statistical Analysis

All data were analyzed statistically using the Statistical Package Software Program for Social Science (Windows version, SPSS Inc., Chicago, IL, USA). Mean and percentage values were used to summarize the baseline characteristics and data outcomes. Data were compared by using a chi-square test. A *p* value of less than 0.05 was considered significant. All the probabilities were evaluated using a two-tailed test. Multivariate binary logistic regression was used to have a model of predictor variables for biofilm formation.

## 3. Results

**Figure 1:**
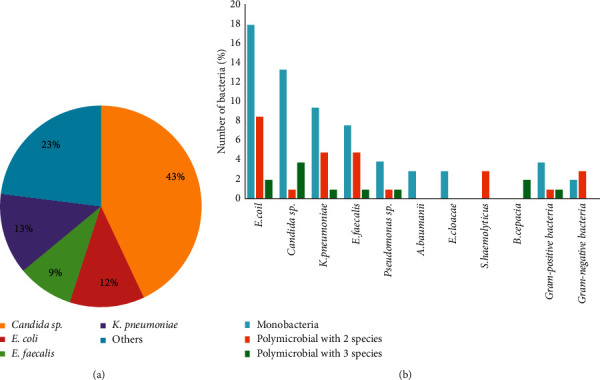
Isolate Distribution in (a) urine culture and (b) catheter culture. *Candida* sp. were found as the dominant microbes in the urine with a percentage of 43%, followed by *E. coli* at 12%, *E. faecalis* at 9%, *K. pneumoniae* at 13%, and other microbes at 23% (1(a)). *E. coli* was the dominant microbes in the catheter as monomicrobial or polymicrobial. *B. cepacia* was also found in the catheter but not found in the urine (1(b)).

During the indwelling catheter and after catheterization, bacteriuria was found in 37 and 22 patients, respectively, among 109 enrolled patients. Positive and negative bacteriuria was found permanently in 9 (8.3%) and 58 patients (53.2%) (Table 2). We also obtained 107 and 43 isolates from the indwelling catheter and urine cultures, respectively. The patients' clinical characteristics are described in Table 3. Microbial growth was observed from 41 (37.26%) of the urine cultures and 85 (78%) of the catheter samples. The majority of the urine isolates were monomicrobial, which included normal microbiota (95.1%) such as *Candida* sp., followed by *Klebsiella pneumoniae, Escherichia coli,* and *Enterococcus faecalis* (Figure 1(a)). On the other hand, each of the catheter cultures had a single microorganism (62.8%) and polymicrobial (26.1% with two microorganisms and 11.1% with three microorganisms). Most of the catheter culture isolates (77.99%) were *Enterobacteriaceae*; while *Burkholderia cepacia* was isolated only from a catheter and never appeared as a single isolate (Figure 1(b)).

**Figure 2:**
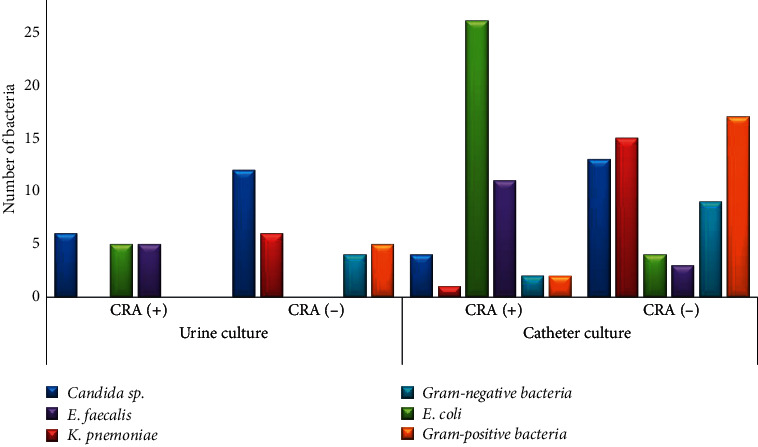
Distribution of isolates between the urine (*n* = 43) and catheter (*n* = 107) cultures according to the CRA test. *E. coli* was the dominant microbial with a positive CRA test in the urine and catheter culture.

The CRA test of urine and catheter isolates was positive in 34.9% of 43 isolates and 40.3% of 107 isolates. The CRA test results showed that 43 out of 107 (40.2%) microorganisms isolated from the catheter were considered biofilm-producing bacteria, in which 57.1% of them were *E .coli*. From urine, we obtained 16 out of 43 (34.9%) microorganisms as biofilm-producing bacteria and most of them were *E. coli* and *E. faecalis* (Figure 2).

Our study revealed that patients with an antibiotic treatment suffered less bacteriuria after catheterization (60% vs. 38.2%). They had a higher conversion rate of bacteriuria from positive to negative (30.7% vs. 4%) compared to those without antibiotics (Table 2).

We also found that not all positive catheter cultures were also positive urine cultures (Table 4). The congruence between urine cultures and catheter cultures, including negative results, was 43%. According to these results, we conclude that if the patient has positive urine culture, the biofilm might have been formed on the catheter.

Table 5 describes the statistical correlation between the risk factors and biofilm formation. The gender and duration of catheterization were statistically significant risk factors of positive biomass results in the indwelling catheter (*p* < 0.05). The female subjects had a significantly higher number of catheters with positive biomass than the male samples (*p* < 0.001). The catheter samples used for five days or more also had a lot more positive biomass results than to the catheters with a shorter indwelling duration (*p*=0.002) (Table 5).

We also performed statistical analysis (chi-square test) to find the correlation between the biofilm-producing bacteria and biofilm formation. Our study results revealed that biofilm-producing bacteria correlated with biofilm formation (*p* < 0.001) (Table 6).

We found that *E. coli* was the most prevalent biofilm-producing bacteria in the catheter and had positive results in the Congo Red Test (Figure 2). Therefore, we characterized the virulence genes, especially PapC, FimA, and SfaS genes, the genes that play a role in surface adhesion, to see the correlation between virulence genes and biofilm formation.

**Figure 3:**
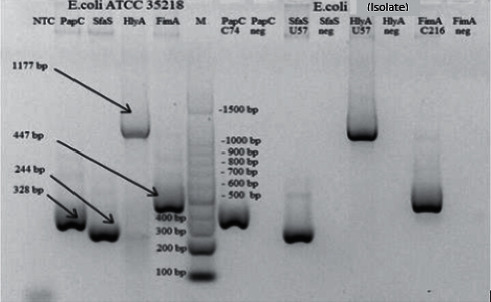
DNA amplification results of *E. coli ATCC 35218* (left) and isolate *E. coli* in this study (right). FimA (447 bp), PapC (328 bp), and SfaS (244 bp) genes.

Characterization was done by a conventional PCR test. 0.  Figure 3 displayed the test results. *E. coli* ATCC 35218 was used as a control because it had all the gene targets.

The proportion of virulence genes FimA, PapC, and SfaS in 30 *E. coli* isolates obtained from catheters were 100%, 60%, and 43%. In 5 *E. coli* isolates obtained from the patients' urine, virulence gene's proportion was 100%, 100%, and 80% (Table 7).

Then the correlation between the virulence genes and biofilm formation was done through a chi-square test. The statistical analysis results showed a significant relationship between the PapC gene and biofilm formation (*p*=0.009). However, there is no significant relationship between FimA or SfaS and biofilm formation (*p*=0.216 and *p*=0.06).

## 4. Discussion

A urinary tract infection (UTI) is one of the most common healthcare-associated infections (HAIs), representing up to 40% of all HAIs [2, 4, 11]. UTI is a *t* common human infectious diseases involving biofilm formation in body tissues or urinary catheter devices [16, 17]. On the other hand, it is also stated that CAUTIs are the most preventable type of HAIs [18]. Therefore, knowing biofilm formation on catheter devices is important to prevent and minimize biofilm formation causing CAUTI. In our study, the most common organisms isolated both from urine and urinary catheter cultures were Gram-negative *Enterobacteriaceae* such as *E. coli* (28.1%), *K. pneumoniae* (15.9%), and *Candida* sp. (17.8%). These organisms are considered CAUTI etiology, and most are endogenous microbiota of the perineum with potential biofilm formation [4, 13, 19–21]. *Enterococcus faecalis* (13.1%) was the predominant Gram-positive organism, which was followed by *Staphylococcus haemolyticus* (2.8%) (Figures 1(a), 1(b), and 2). Other studies have also reported a similar diversity of microorganisms found on a catheter [12, 22–27]. Various literature has described that the organisms may ascend from a catheter by direct inoculation, whether at the time of catheter insertion or when the system was opened for changing the urinary bag, or by migration from perineum colonization to the external surface of the catheter [20, 28–30]. There were no significant differences regarding organisms isolated between urine and urinary catheter cultures, and the congruency was 43%. It indicates that urinary catheter insertion might increase UTI risk [1, 31, 32].

About 37.2% of catheter cultures were polymicrobial, and 72.2% had a catheterization duration of ≥5 days. The duration of catheterization is one of the main roles in polymicrobial infection. In the long-term catheterization (several weeks and months), a polymicrobial infection is inevitable [31, 33]. Our study also found the same facts that polymicrobial on a catheter were relevant to the duration of catheterization (mean 5.6 ± 2.1). Interestingly, we found that *Burkholderia cepacia* was always present and coexisting with other microbes. *Burkholderia cepacia* is known as an opportunistic pathogen in immunocompromised patients with cystic fibrosis. It can cause infections in other sites such as the skin, bloodstream, and urinary tract [34, 35]. The microbial interactions between uncommon and pathogenic bacteria in influencing the UTI remain unclear, but the potential implications like antagonistic or synergistic interactions have received more attention [31]. As the most prevalent causative agent of polymicrobial biofilms, *E. coli* showed more resistance to antibiotics and were more invasive in vitro epithelial cell infection studies [36]. In fact, a bacterial metabolism seems to contribute to the persistence and pathogenesis of bacteria within biofilms as much as the virulence abilities [37].

*Candida* appears as predominant yeast, both in urine and catheter cultures. Some *Candida* sp. was found as polymicrobial with other bacteria such as *E. coli*. They could exhibit cooperative interactions in UTI-related settings [31]. *E. coli* can enhance the adhesion of *C. albicans* to the bladder mucosa, thus increasing the chance of fungal UTI [38]. It corresponds with the assumption that *Candida* sp. findings are usually related to opportunistic colonization without the urgent need for antifungal treatment [17]. However, clinicians need to be cautious about critically ill or immunocompromised patients since candiduria could develop into a UTI or even systemic infection in this population [17, 39].

Several studies on urogenital microbiomes suggest that Gram-positive microorganisms such as *Lactobacillus* sp., *Streptococcus* sp., *Staphylococcus* sp., and *Corynebacterium* sp. are the common microorganisms isolated from urogenital area samples in patients with no infection [40–43]. The normal flora of the urogenital, especially *Lactobacillus* sp., has a role in maintaining the balance of microbes and preventing the colonization of potential pathogens [42–44]. Normal flora could be diminished by several factors, such as gender, age, and antibiotic usage. In our study, 67.8% of the patients had been exposed to mostly broad-spectrum antibiotics. They potentially disturbed the balance of urogenital microbiomes and promoted the growth of Gram-negative bacteria, including the biofilm-producing bacteria. However, there was no statistically significant correlation between antibiotic usage and biofilm formation (*p*=0.338) (Table 5). Therefore, antibiotic usage might not decrease biofilm formation.

About 68.8% of the patients in our study had taken antibiotic medication prior to urinary catheterization. We found that the usage of systemic antibiotics reduced the risk of catheter-associated bacteriuria (Table 5). The lack of systemic antimicrobial agents has also been found to increase the risk of catheter-associated bacteriuria in several studies [45, 46]. However, systemic antimicrobial prophylaxis should not be routinely advised in patients with short-term or long-term catheterization, including patients who undergo surgical procedures, due to great concern about antimicrobial resistance [26]. In asymptomatic catheter-associated bacteriuria, prophylactic antimicrobials may postpone bacteriuria. However, they do not prevent further complications and can lead to a reservoir of antibiotic-resistant organisms within a hospital [4, 26, 28, 47]. Catheter-associated bacteriuria is difficult to eradicate as long as the catheter remains in place due to the presence of biofilms, which enhances an organism's ability to colonize a urinary catheter and protect them from antimicrobials and host defense mechanisms. Our study also considered that antimicrobial usage did not correlate with biofilm formation, although it could reduce the bacteriuria. We found that the percentage of positive catheter cultures was higher than urine cultures (78% vs. 37.26%). It means that there was high colonization of microbiota on the device during catheterization, and it indicates that catheter colonization was made prior to detectable bacteriuria. In addition, a viable but nonculturable state of some microorganisms and antibiotic administration might decrease the number of positive urine cultures [31, 48]. This microbiota colonization is the first step to biofilm formation [12, 31, 49, 50]. Since a catheter-associated urinary tract infection is rarely symptomatic and urine cultures are not always positive, although they have already had microbe on the biofilm, these reasons might increase the urosepsis risk [4, 11]. However, it may confirm the previous report that bacteriuria in patients with an indwelling urinary catheter is due to bacteria ascending from within biofilm formed on the catheter surface [1, 21, 51, 52].

In Table 6, the statistical analysis shows the correlation between biofilm-producing microbes and biofilm formation (*p* < 0.001). We assume that biofilm-producing microbes could indicate biofilm growth in a urethral catheter (95% of the biofilm-producing bacteria formed the biofilm on the catheter). Precautions need to be taken by clinicians when they find these biofilm-producing bacteria in urine cultures from their patients, as these bacteria might form the biofilm in either the catheter or the tissues of the urinary tract that can be a part of CAUTI pathogenesis [2, 11, 20, 28, 53]. Our study revealed that most of the biofilm-producing bacteria were *C. albicans*, *E. coli*, and *E. faecalis*, which is similar to other studies' results [54].

Gender is widely accepted as a risk factor for urinary tract infection. In female geriatric patients, the *Lactobacillus* sp. population is decreasing, presumably because of an estrogen decrease and an increase in the pH of the urogenital area, which allows for the colonization of uropathogens [44]. Some studies indicate that women have a higher risk of bacteriuria compared to men [28]. Women in general also develop four times more urinary tract infections than men because of anatomic differences, including a shorter urethra and normal vaginal flora that colonize the external urethra [2, 19, 30]. We measured the presence of intraluminal and extraluminal catheter biofilm and discovered a significant correlation between gender and positive biomass findings (*p* < 0.001). For samples taken from a catheter, female patients gave more positive biomass results than male patients.

In our study, catheterization for five days and more has been a significant risk factor for biofilm formation, and 84% of 67 samples formed the biofilm. The statistical analysis also showed a significant correlation between the catheterization duration and biofilm formation in the catheter. The biofilm in our study was detected in an acute setting (a mean of 5.6 ± 2.1 days). Macià et al. suggested that when the duration of catheterization is extended, the infection may be polymicrobial. When patients are also receiving antibiotics, the isolation of multidrug-resistant Gram-negative bacilli is relatively common [55]. Our study has demonstrated that the duration of catheterization of fewer than five days also had an opportunity to form a biofilm (57%). This finding suggests that for patients with short-term catheterization, we still have to consider the risk of biofilm formation for those with risks [5].

Age and diabetes mellitus status are known as risk factors of CAUTI. However, our study found that the correlation between biofilm and these factors was statistically insignificant. The multivariables of gender, duration, and bacteriuria prior to catheterization are the predictor variables of the model for biofilm formation. Therefore, they may increase the risk of getting CAUTI. However, we also found that bacteriuria prior to catheterization as a single variable was statistically insignificant in affecting biofilm formation (*p*=0.078). Therefore, we assume that the biofilm formation process continued despite no appearance of bacteria in the urinary tract before catheterization.

We also characterized the virulence genes of *E. coli* as dominant bacteria that we found in our study to show the relationship between virulence genes and the ability to form a biofilm. Most of the biofilm-producing *E. coli* in this study were obtained from urine samples and catheter tip samples with FimA and PapC genes (100% vs. 100% and 60% vs. 100%). The proportion of bacteria that have the FimA gene and PapC gene is similar to other studies' results [56]. Fatahi et al. reported that the FimA gene's prevalence was 94%, and the PapC gene was 43% [57]. Another study conducted in Iran also found the proportion of the PapC gene and SfaS gene was 74% and 54% in UPEC isolated from UTI patients [27]. Naves et al. [58], Soto et al. [59], and Tarchouna et al. [6] showed that biofilm-producing *E. coli* have a PapC gene commonly found in bacteria isolated in UTI patients. Another study also reported that the PapC gene was found frequently in biofilm-producing *E. coli* [6, 57]. The previous studies were similar to the results in this study, where biofilm-producing *E. coli* have the FimA gene (100%), while the PapC gene (71%) was more the other way around (29%) (*p*=0.009). Therefore, it means that the PapC gene influences biofilm formation. However, the expression of the SfaS gene in biofilm-producing *E. coli* was lower (6%) than in non-biofilm-producing *E. coli* (93.3%). It shows that the SfaS gene does not impact biofilm formation in the catheter (*p*=0.952). In general, this study showed that PapC and FimA genes have a relationship with biofilm formation in *E. coli*.

## 5. Conclusion

The most frequently isolated microorganisms from a urinary catheter are *Escherichia coli* (28.1%) followed by *Candida* sp. (17.8%), *Klebsiella pneumoniae* (15.9%), and *Enterococcus faecalis* (13.1%). Biofilm-producing bacteria are found in 40% of isolates. *Escherichia coli* is the most common finding with the PapC gene as a virulence factor that impacts biofilm formation. The risk factors correlated with biofilm growth in the urethral catheter are female gender, catheterization duration of more than five days, and bacteriuria before catheterization. The positive findings of biofilm-producing bacteria in the urinary catheter could be an indicator of biofilm formation. These findings still raise several questions to be answered, such as the clinical impacts of the polymicrobial biofilm-producing bacteria findings in the urinary catheter and how they might affect the disease outcome. We also note that many uncommon bacteria were undetected from the bacterial culture due to the lack of media and kits in hospitals.

## Figures and Tables

**Table 1:** Temperature of the PCR condition.

Gene	Predenaturation (°C/t)	Denaturation (°C/t)	Annealing (°C/t)	Extension (°C/t)	Final extension (°C/t)	Cycle
PapC	95/3′	95/30″	62/30″	72/40″	72/5′	35
FimA	95/5′	95/30″	62/30″	72/30″	72/5′	35
SfaS	94/5′	94/30″	60/30″	72/25″	72/5′	35

**Table 2:** Bacteriuria status on pre- and posturethral catheterization with the usage of antibiotics.

Bacteriuria pre- and posturethral catheterization	Usage of antibiotics (*n*, %)	No usage of antibiotics (*n*, %)
Permanently positive	4 (5.3)	5 (14.7)
Permanently negative	45 (60.8)	13 (38.2)
Conversion from positive to negative	23 (30.7)	5 (14.7)
Conversion from negative to positive	3 (4.0)	11 (32.4)
Total	75 (100)	34 (100)

**Table 3:** Baseline characteristics of 109 patient respondents.

Characteristics	Number (%)
Age (18–88 years)	Mean: 56.89 ± 17.28
<60 years	58 (53.2)
≥60 years	51 (46.8)
Sex	
Male	42 (38.53)
Female	67 (61.47)
Duration of catheterization (days)	Mean: 5.6 ± 2.1
<5 days	43 (39.4)
≥5 days	66 (60.6)
Patients with diabetes mellitus	24 (22)
Bacteriuria detected before catheterization	37 (33.9)
Patients with antibiotic exposure prior to catheterization	75 (68.8)

**Table 4:** Comparison of the results of urine cultures and catheter cultures.

	Catheter cultures (+) (*n*, %)	Catheter cultures (−) (*n*, %)
Urine cultures (+)	41 (48)	0 (0)
Urine cultures (−)	44 (52)	24 (100)

**Table 5:** Risk factor distribution.

No.	Risk factor	Biofilm formation (*n*, %)		*p* value (*p* < 0.05)	OR	95% CI
		Positive	Negative			
1	Gender					
	Male	21 (50)	21 (50)	≤0.001	0.136	0.052–0.352
	Female	59 (88)	8 (12)			
2	Age					
	≥60 y.o.	40 (80)	10 (20)	0.151	1.900	0.786–4.591
	<60 y.o.	40 (68)	19 (32)			
3	Diabetes mellitus					
	Positive	17 (71)	7 (29)	0.748	0.848	0.310–2.317
	Negative	63 (74)	22 (26)			
4	Antibiotics					
	Without AB	27 (79)	7 (21)	0.338	1.601	0.608–4.218
	With AB	53 (71)	22 (29)			
5	Duration					
	≥5 days	56 (84)	11 (16)	0.002	0.262	0.108–0.637
	<5 days	24 (57)	18 (43)			
6	Bacteriuria before catheterization					
	Positive	31 (84)	6 (16)	0.078	2.425	0.888–6.624
	Negative	49 (68)	23 (32)			

**Table 6:** Relationship between the biofilm-producing bacteria and the biofilm formation.

No.	Parameter	Biomass (*n*, %)		*p* value	OD	95% CI
		Positive	Negative			
1	Biofilm-producing bacteria	40 (95)	2 (5)	≤0.001	0.074	0.017–0.333
2	Non-biofilm-producing bacteria	40 (60)	27 (40)			

**Table 7:** Proportion of biofilm-forming *E. coli* virulence genes.

Virulence gene targets	*E. coli* isolate from a catheter		*E. coli* isolate from urine
	CRA pos	CRA neg	CRA pos
*FimA*			
Positive	26	4	5
Negative	0	0	0

*SfaS*			
Positive	11	1	4
Negative	15	3	1

*PapC*			
Positive	17	2	1
Negative	9	2	4

## Data Availability

The datasets used and/or analyzed during the current study are available from the corresponding author upon reasonable request.

## Abbreviations

CAUTI: Catheter -related urinary tract infection.
